# A Novel Magnetic Actuation Scheme to Disaggregate Nanoparticles and Enhance Passage across the Blood–Brain Barrier

**DOI:** 10.3390/nano8010003

**Published:** 2017-12-22

**Authors:** Ali Kafash Hoshiar, Tuan-Anh Le, Faiz Ul Amin, Myeong Ok Kim, Jungwon Yoon

**Affiliations:** 1 Faculty of Industrial and Mechanical Engineering, Islamic Azad University, Qazvin Branch, Qazvin 34199-15195, Iran; Hoshiar@Qiau.ac.ir; 2 School of Integrated Technology, Gwangju Institute of Science and Technology, 123 Cheomdangwagi-ro, Buk-gu, Gwangju 61005, Korea; tuananhtbbk@gmail.com; 3 Department of Biology and Applied Life Science, Gyeongsang National University, Jinju 660-701, Korea; faizgnu@gmail.com (F.U.A.); mokim@gnu.ac.kr (M.O.K.)

**Keywords:** asymmetrical discontinuous field function, blood–brain barrier (BBB), magnetic drug delivery, magnetic nanoparticles, aggregation

## Abstract

The blood–brain barrier (BBB) hinders drug delivery to the brain. Despite various efforts to develop preprogramed actuation schemes for magnetic drug delivery, the unmodeled aggregation phenomenon limits drug delivery performance. This paper proposes a novel scheme with an aggregation model for a feed-forward magnetic actuation design. A simulation platform for aggregated particle delivery is developed and an actuation scheme is proposed to deliver aggregated magnetic nanoparticles (MNPs) using a discontinuous asymmetrical magnetic actuation. The experimental results with a Y-shaped channel indicated the success of the proposed scheme in steering and disaggregation. The delivery performance of the developed scheme was examined using a realistic, three-dimensional (3D) vessel simulation. Furthermore, the proposed scheme enhanced the transport and uptake of MNPs across the BBB in mice. The scheme presented here facilitates the passage of particles across the BBB to the brain using an electromagnetic actuation scheme.

## 1. Introduction

In recent years, many nanorobotic systems have emerged for studies in biology [1,2,3,4,5], and developments in magnetic nanoparticles (MNPs) for biomedical applications have considerably exceeded expectations, as the versatile natural properties of MNPs facilitate biological applications such as drug delivery. Functionalized MNPs have showed encouraging results in crossing the blood–brain barrier (BBB) [6,7]. Magnetic drug delivery (MDD) can be used to increase drug uptake. In MDD, the drug is added to MNPs, which are injected into a blood vessel and circulate throughout the vasculature. An external magnetic field is next applied to achieve the optimal concentration of drug-loaded particles in the desired location [8,9].

MNPs have a myriad of applications as therapeutic and diagnostic agents. Recent studies of MNPs for MDD applications have revealed their magnetic properties, biocompatibility, and toxicity [10]. MNPs have unique optical properties suitable for in vivo tracking and are capable of delivering drugs to brain cells [11]. Drug-conjugated MNPs are used for drug delivery [12,13] by applying an external magnetic field to a location of interest in the body.

Although drug delivery using a magnetic field has been around for decades [14], recent developments have made MNPs feasible for MDD. Studies on MDD have focused on simulation and analysis of the captured and retained particles using a constant external magnetic field. Numerical simulations of blood flow and MNP distribution in a realistic brain vessel have demonstrated that MDD significantly increases particle capture [15]. The particle size, type and coating, which influence capture efficiency, were studied in a computer simulation; the particle retention decreased with an increasing particle size [16]. However, particle sticking and aggregation were neglected in these simulations.

The concentration of MNPs under a constant magnetic field using a Y-shaped bifurcation has been reported [17], and the aggregation of MNPs under a constant magnetic field has been examined experimentally [18]. MNPs reportedly stick to vessel walls because of the low flow velocity [19]. The steering of aggregated microparticles under a constant magnetic field in a Y-shaped channel results in the accumulation of aggregates at the bifurcation [20]. A constant magnetic field is used to facilitate passage across the BBB, which is mediated by endocytosis [21]. Real-time in vivo monitoring of drug delivery using a constant magnetic field has been evaluated [22]. Moreover, after crossing the BBB under the guidance of a uniform magnetic field, MNPs formed rod-shaped aggregates [23]. Although in [18], sticking was reduced by changing the shape of the magnet, this is not a general solution to sticking; indeed, despite successful passage across the BBB, sticking and aggregation were not considered in [21].

We have resolved particle sticking by simulating intentional changes in the magnetic field direction [24]. The use of dynamic magnetic actuation (change in field direction) to reduce aggregation was investigated in [25]. The findings in [26] showed that dynamic actuation with a pulse-shaped magnetic field using permanent magnets improves passage through the BBB. To evaluate drug uptake in the brain, time-varying dynamic magnetic actuation was evaluated in the brains of mice. In the absence of a magnetic field, no nanoparticles (NPs) were found in the brain [27]. Using the magnetic field function (FF), however, the rate of BBB passage and drug uptake increased significantly [27]. Despite the acceptable performance in vivo, aggregation in a magnetic field was not modeled. To improve dynamic actuation for BBB passage, particle aggregation should be modeled.

Cluster structure and aggregation were previously evaluated numerically in a two-dimensional (2D) platform [28]. Vartholomeos and Mavroidis developed a simulation platform to aggregate MNPs and increase the magnetic force in a simulation [29]. A computational platform was designed to assess the guidance of aggregated particles under a constant magnetic field [30]. A simulation platform was also developed to deliver aggregated particles under a dynamic magnetic field in a Y-shaped channel [31]. However, a discontinuous magnetic actuation scheme that minimizes aggregation and increases the rate of BBB passage has not been reported to date.

**Figure 1:**
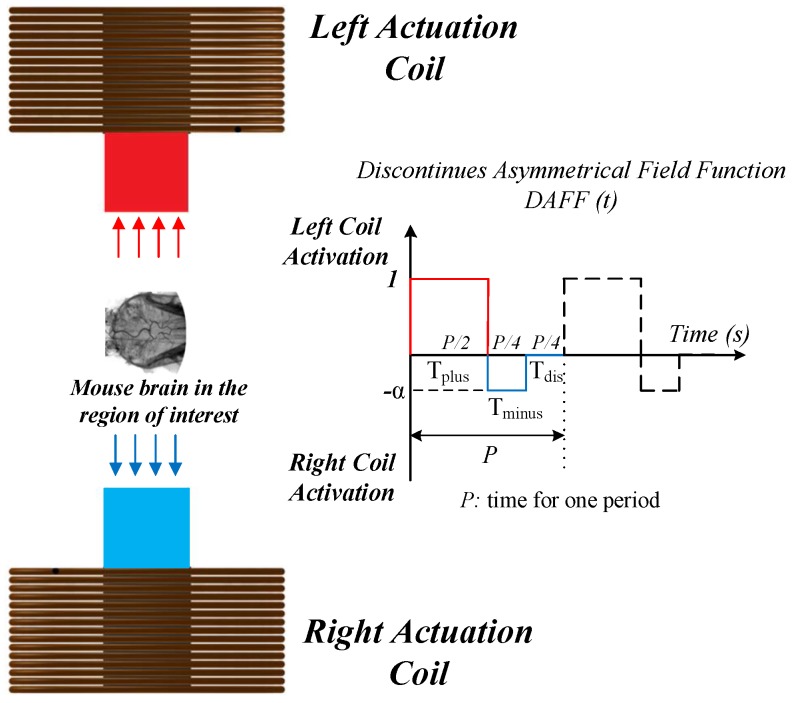
Schematic of the discontinuous magnetic actuation system for drug delivery and blood–brain barrier (BBB) passage using the proposed discontinuous asymmetrical field function (DAFF).

In this scheme, the NPs are injected into a vein and circulate through the vasculature. We propose a novel discontinuous asymmetrical dynamic actuation scheme to change the magnetic field and deliver MNPs. This method is aimed at facilitating BBB passage by minimizing aggregation through the insertion of a deactivation time ($T_{dis}$) between each cycle, as shown in Figure 1. A simulation platform is utilized to assess the delivery of aggregated particles under the dynamic actuation scheme. A discontinuous asymmetrical field function (*DAFF*(*t*)), which generates a discontinuous unequal alternating magnetic gradient, enables changes in the field direction to guide MNPs. The functionality and performance of this approach in terms of particle sticking were evaluated in a realistic three-dimensional (3D) simulation of a vessel. The proposed design improved BBB passage by MNPs and decreased the size of aggregates after BBB passage.

This paper is organized as follows: In Section 2, the computational model for particle guidance is developed, the concept and design of the DAFF are introduced, and the DAFF is studied for steering MNPs in a Y-shaped channel. In addition, the effects of the DAFF on particle sticking have been investigated in a realistic 3D vessel simulation. Finally, an in vivo evaluation of the passage through the BBB by MNPs is presented. Section 3 presents the setup of the experiments. We conclude that the proposed scheme increases the rate of BBB passage by MNPs.

## 2. Results and Discussion

### 2.1. Governing Dynamic Forces in MDD

**Figure 2:**
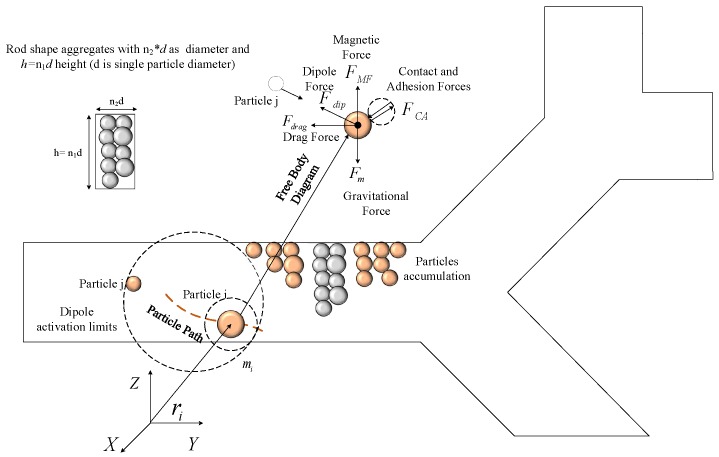
A particle *i* inside the vessel is considered; the effective forces are shown in the free-body diagram and the geometry of the rod-shape aggregates is illustrated.

To fully understand MNP aggregation, the forces governing MNP steering are modeled in this section. Many parameters presented in this section are used throughout the manuscript; any changes in them are stated. The forces depicted in Figure 2 are considered, and the Newtonian dynamic model is used:

(1) $$m_{i}\frac{dv_{i}}{dt}=F_{MF}+F_{\mathrm{dip}}+F_{\mathrm{drag}}+F_{CA}+F_{m}$$

where index *i* indicates particle *i*, $m_{i}$ is the particle mass, $v_{i}$ is the particle velocity, $F_{MF}$ is the magnetic force, $F_{dip}$ is the dipole force, $F_{drag}$ is the hydrodynamic drag force, $F_{m}$ is the gravitational force (gravity and buoyancy), and $F_{CA}$ is the contact–adhesive force. To use Newtonian mechanics, particles are considered to be large enough to exclude the effect of Brownian motion [29,32].

The magnetic force is the actuation force used for steering. MNPs exhibit almost hysteresis-free behavior. If the permeability in the medium satisfies the relation $\mu_{1}=\mu_{0}$ ($\mu_{0}$ is the permeability of the free space) and, considering the magnetic polarization (*M*) as a function of magnetic intensity (*H*), which has a finite limit of $M_{sat}$, if the magnetic field is considered large enough to create the finite value $M_{sat}$ and *V* is considered to be the volume of the rod-shaped aggregates, then the magnetic force can be modeled as

(2) $$F_{MF}=V\mu_{1}M_{\mathrm{sat}}\cdot \nabla H_{f}$$

The rod-shaped aggregates have a diameter of $n_{2}d$ and a height of $n_{1}d$, with *d* being the diameter of a single MNP; $n_{1}$ and $n_{2}$ are the number of particles in the aggregate (the rod-shaped aggregates are shown in Figure 2). The aggregate volume is represented as $\frac{\pi }{4}n_{1}n_{2}^{2}d^{3}$.

$F_{dip}$ is the dipole force, which plays a major role in keeping the particles together. The dipole force is modeled as

$$F_{dip}=\frac{3\mu_{1}m_{i}m_{j}}{4\pi r_{ij}^{4}}(r_{ij}\left(m_{i}\cdot m_{j}\right)$$

(3) $$+m_{i}\left(r_{ij}\cdot m_{j}\right)+m_{j}\left(r_{ij}\cdot m_{i}\right)-5r_{ji}\left(r_{ji}\cdot m_{i}\right)\left(r_{ji}\cdot m_{j}\right)$$

where $\mu_{1}$ is the magnetic permeability of the medium, $m_{i}$ and $m_{j}$ are the magnetic moments of the *i*th and *j*th particles and $r_{ij}$ is the distance between particles.

During aggregation, the dipole force has two main effects: an initial negligible contribution to magnetic intensity (*H*), and a major influence on particle–particle sticking. To model the magnetic moment, a system of coupled equations must be solved [29,30]. The total magnetic intensity for the particle of interest is given as

(4) $$H=H_{\mathrm{ext}}+\sum_{j}^{N}H_{\mathrm{dip}}$$

where $H_{ext}$ is the external magnetic intensity, and sum is the accumulated effect of other particles.

The drag (hydrodynamic) force on the particles based on Stokes law is

(5) $$F_{\mathrm{drag}}=-3\pi \eta d\left(v_{p}-v_{f}\right)$$

where $v_{p}$ and $v_{f}$ are the particle and fluid velocities, respectively; *d* is the particle diameter; and η is the fluid viscosity.

The gravitational force is yielded by gravity and buoyancy forces as follows:

(6) $$F_{m}=\frac{1}{6}\pi d^{3}\left(\rho_{p}-\rho_{b}\right)G$$

where *d* is the particle diameter, and $\rho_{p}$ and $\rho_{b}$ are the particle and blood densities, respectively.

The contact–adhesive forces are generated by particle–particle or particle–surface collisions. The Hertzian contact model can be expressed as

(7) $$F_{c}=k\delta^{\frac{3}{2}}\overset{\mathrm{If}}{↔}P_{dis}<R_{i}+R_{j}$$

where $P_{dis}$ is the particle–particle distance, $R_{i}$ is the *i*th particle radius, $R_{j}$ is the *j*th particle radius, *k* is the spring constant, and δ is the deformation.

The adhesive force is also modeled as

(8) $$F_{Ad}=\tau \pi {\left(\frac{3F_{c}d}{8E^{*}}\right)}^{\frac{2}{3}}$$

where τ is the adhesive energy (a constant parameter), *d* is the equal diameter $\left(d=2*\frac{R_{1}R_{2}}{R_{1}+R_{2}}\right)$, $F_{c}$ is the contact force, and $E^{*}$ is the equal elasticity module. The opposite nature of the contact force (separation) and adhesion force (connection) creates the contact–adhesive force, which is represented as

(9) $$F_{CA}=F_{Ad}+F_{c}$$

The trajectories of the MNPs can be determined by incorporating the forces in Equation (1).

### 2.2. Simulation Platform for Steering Aggregated MNPs in Bifurcations

A Y-shaped channel that resembles a bifurcation was used in the simulation. The Y-shaped channel consists of one inlet and two outlets of constant diameter. A steady creeping flow enters through the inlet and exits via the outlets. Aggregation is considered to occur near the inner boundary of the vessel. Initially, the particles attract each other as a result of dipole effects. The contact–adhesive force balances this effect and mediates MNP aggregation. The magnetic force acts as a body force and moves the particle toward the direction of application; the drag force resists this movement. These forces are incorporated into the governing dynamics (Equation (1)), and a system of ordinary differential equations (ODEs) is formed.

In this simulation, 225 particles (800 nm diameter) were used, and a system of 900 ODEs was solved at each time-step. The ODE system is numerically solved using the Runge Kutta method. The magnetic and drag forces govern the dynamics of the movement of particles inside the vessel. The magnitude of the magnetic forces varies with the number of aggregated particles in a rod. Therefore, the particle velocity varies according to the aggregate size. The number of particles in the aggregates determines the velocity; therefore, the aggregate size was used in the simulation to match the experimental data [31,33].

**Figure 3:**
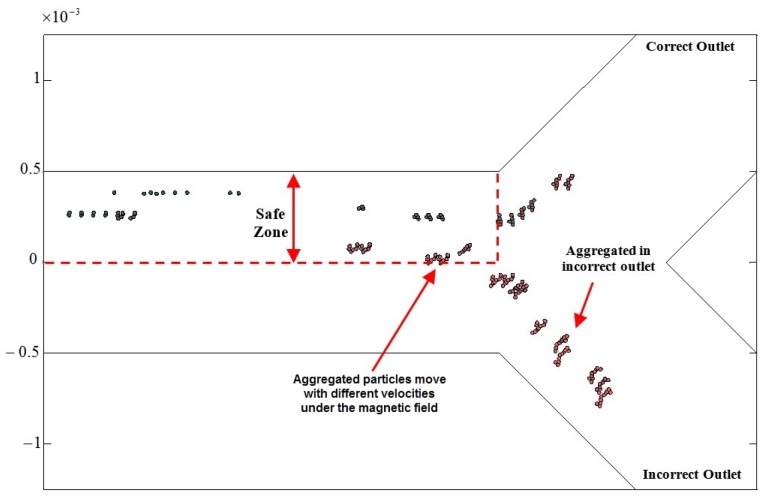
Steering aggregated magnetic nanoparticles (MNPs) in a Y-shaped channel using magnetic actuation.

Figure 3 shows a simulation of MNP aggregation within the Y-shaped channel. The rod-shaped aggregates move on the basis of the magnetic actuation at different velocities and reach the bifurcation. Using this simulation platform, which considers the physical parameters in Table 1, the guidance could be evaluated by computing the number of particles that reached the correct outlet. It is assumed that particles that remain inside the safe zone will be guided to the correct outlet [33]. The safe zone is the distance between the vessel boundary of the correct outlet and the mid-vessel line (Figure 3). A high percentage of particles reaching the correct outlet reflects the delivery performance of the MNPs by the magnetic field.

#### 2.2.1. The Influential Parameters in Targeting Performance

Three coefficients were introduced to investigate the effect of aggregation on the guidance. The guidance performance in a Y-shaped bifurcation depends on the vessel elongation ratio ($R_{ve}$) for the vessel geometry, the normal exit time ($T_{c}$) as an environmental condition, and the force factor ($R_{f}$) [24].

The normal exit time represented by $T_{c}$ shows the influence of vessel elongation and flow velocity. This factor represents the minimum time needed for the aggregated particles to reach the bifurcation point. In a vascular network, the blood-flow velocity varies between a few millimeters per second in capillaries to a few centimeters per second in arteries [20]. The normal exit time is shown in Equation (10). The designed actuation should be robust to these changes and should be able to safely guide the particles to the desired outlet. To study this effect, the vessel nominal length was considered to be 10 mm and the normal exit time varied on the basis of the information in Table 2.

(10) $$T_{c}=(\frac{L_{v}}{V_{b}})$$

where $L_{v}$ is the vessel length and $V_{b}$ is the flow velocity.

The diameter and length of blood vessels vary; this is represented by the vessel elongation ratio $R_{ve}$, which is considered to a dimensionless factor comprising the vessel-length-to-diameter ratio. To study this parameter, the vessel nominal length was considered to be 10 mm and the $R_{ve}$ was changed on the basis of the information in Table 2. The vessel elongation factor is

(11) $$R_{ve}=(\frac{L_{v}}{D_{v}})$$

where $L_{v}$ is the vessel length and $D_{v}$ is the vessel diameter.

The magnetic actuation force is mainly affected by two parameters: the magnitude of the magnetic field gradient and the particle size. To evaluate the effect of the actuation force, the force factor is defined as

(12) $$R_{f}=(H_{g}d^{3})$$

where $H_{g}$ is the magnetic gradient and *d* is the particle diameter. The particle diameter was considered to have a mean value of 800 nm, and the force factor used in the simulations is presented in Table 2.

The ability of a constant-direction magnetic field to steer MNPs is reportedly limited by particles sticking to the vessel. This can be solved by alternating the dynamic magnetic actuation [24]. A field function (FF(t)), which is a unitless multiplier, is proposed to change the direction of the magnetic field by activating the coils sequentially [24]. FF(t) for all values of *t* is defined as

(13) −1≤FF(t)≤1

Here, the minus sign indicates the right coil (direction of incorrect outlet), and the plus sign indicates the left coil (direction of the correct outlet) (Figure 1). This function defines the magnetic field and has two properties: the frequency (Hz) and duty ratio (dimensionless) of activation time. The ratio of the activation time for the coil in the direction of the correct outlet to the activation time for the coil in the direction of the incorrect outlet was considered to be 3 to 1. Different frequencies were considered for the simulation of MNP guidance [24]. Using FF(t), the alternating gradient field is defined as

(14) $$\nabla H_{f}=FF\left(t\right)\nabla H$$

Consequently, the actuation force is designed as

(15) $$F_{MF}=FF\left(t\right)VM_{\mathrm{sat}}\cdot \nabla H$$

**Figure 4:**
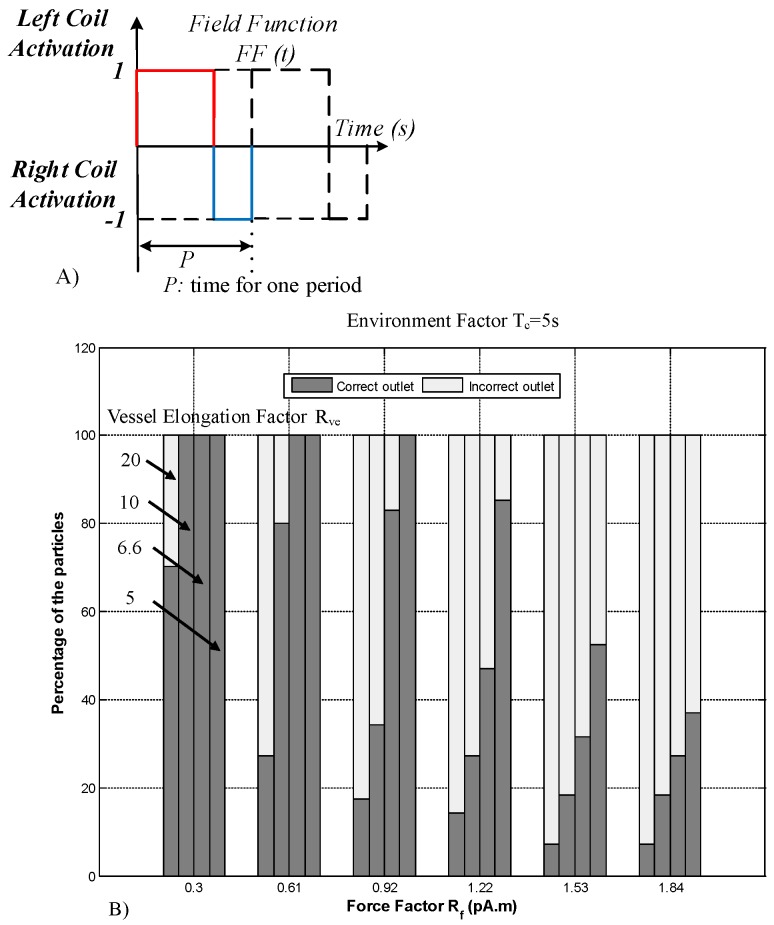
Simulation results for aggregated particle guidance. (**A**) The field function for magnetic actuation. (**B**) The delivery performance in a simulation of a Y-shaped vessel with $d_{v}=1$ mm, $L_{v}=10$ mm, $T_{n}=5$ s and field function (FF)(t) with 6 A and duty cycle of 0.5 Hz.

The frequency of FF(t) (Figure 4A) was considered to be 0.5 Hz, which is the best frequency suggested in [24]. However, the previous simulation did not consider the effects of the aggregation of MNPs within the magnetic field. A previously developed computational platform (Figure 3) with aggregation modeling was used to study the guidance performance of aggregated MNPs. The number of particles reaching the correct outlet was calculated. The simulation results for aggregated particle guidance for all the conditions in Table 2 are shown in Figure 4.

The simulation results reveal two patterns of aggregated particle guidance with FF(t) (Figure 4). By increasing the force factor (from $R_{f}$ of 0.3 to 1.84), the success rate decreased in all cases. This is because when a higher force is applied, more particles leave the safe zone, and thus the guidance performance is decreased by the increase in the force factor. Therefore, a clear trend of deterioration is observable for higher force factors. Moreover, a decrease in the vessel elongation factor results in an increase in the rate of successful guidance (Figure 4). As the vessel elongation factor increases, the time needed for the particles to leave the safe zone increases. In this simulation, as the particles were considered to be aggregated, the normal exit time was not particularly influential.

As illustrated in Figure 4, the delivery performance of FF(t) is sensitive to parameter changes. Moreover, the magnetic actuation (FF(t)) proposed in [24] does not include the effects of aggregation, and it generates large aggregates that hinder BBB crossing. Therefore, this paper uses a discontinuous asymmetrical field function to solve these issues.

#### 2.2.2. The DAFF Design

The magnetic actuation scheme should be modified to reduce the adverse effects of aggregation. Therefore, we propose a DAFF to solve the aggregation issue. The DAFF is a unitless multiplier with an asymmetric ratio of α and a magnitude of 1. The asymmetry ratio α is used to handle the aggregation effect and keep particles inside the safe zone (illustrated in Figure 3). The DAFF also alternates the magnitude of the magnetic field sequentially. The DAFF(t) is illustrated in Figure 1 and for all values of *t* is defined as

(16) −α≤DAFF(t)≤1α≤1

Here, the minus sign indicates the right coil (to the incorrect outlet), and the plus sign indicates the left coil (to the correct outlet), as illustrated in Figure 1.

In the absence of a magnetic force, the aggregated particles disaggregate as a result of the effects of Brownian and drag forces. $T_{dis}$ is the time of discontinuity, during which both coils are inactive, considered in the DAFF. The DAFF is defined by the activation ratio, discontinuity time ($T_{dis}$), frequency, and asymmetry ratio (α). The DAFF has an activation ratio of 2 to 1 (for the coils), and $T_{dis}$ is considered to be equal to $T_{minus}$ (Figure 1). The magnetic actuation force is introduced as

(17) $$F_{MF}=DAFF\left(t\right)VM_{\mathrm{sat}}\cdot \nabla H$$

**Figure 5:**
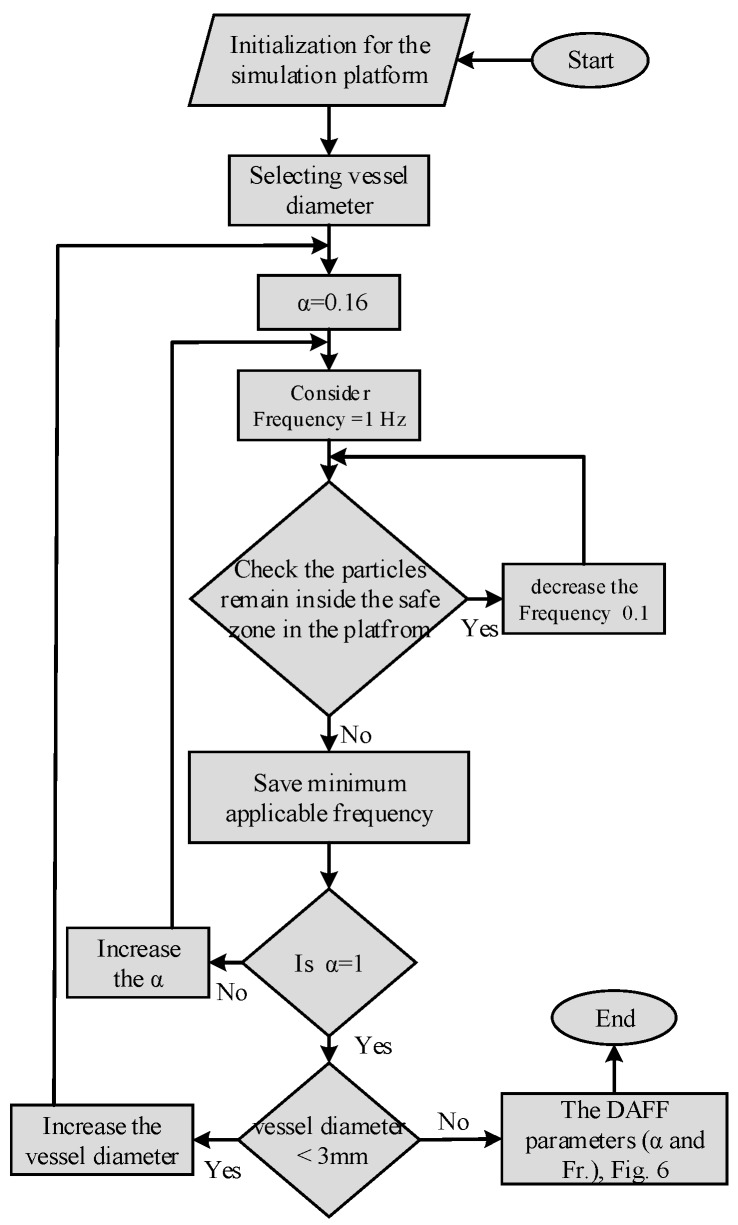
Flowchart of the frequency and α in the discontinuous asymmetrical field function (DAFF).

The design objective here is to determine the magnitude of α (asymmetry ratio) and frequency so that all particles are retained inside the safe zone (Figure 3). With the designed frequency and asymmetry ratio, the particles will remain in the safe zone and can reach the correct outlet. Utilizing the developed computational platform, the frequency and α have been obtained to satisfy the design objective. The simulation flowchart for the DAFF is shown in Figure 5.

The currents applied to the coils are 1, 2, 3, 4, 5, and 6 A [27]. Therefore, the asymmetry ratio α is considered to be 0.16, 0.33, 0.5, 0.66, 0.83, and 1, respectively. For a predefined bifurcation geometry with a diameter of 1 mm and length of 10 mm, initially, the asymmetry ratio α is considered to be 0.16. In step 1, the actuation frequency is considered to be 1 Hz; then, the simulation platform is used to verify that all the particles remain in the safe zone. If all the particles remain in the safe zone, the frequency is decreased in 0.1 Hz increments. This cycle repeats unless the particles exit the safe zone. The minimum frequency that retains all aggregates inside the safe zone is obtained. In step 2, the asymmetry ratio α is increased; this process is repeated for different values of α (0.16, 0.33, 0.5, 0.66, 0.83, and 1). In step 3, the vessel diameter is changed (2 and 3 mm), and the above process is repeated to determine the adequate frequency for each vessel diameter. The flowchart in Figure 5 shows the process of determining the adequate frequency according to the asymmetry ratio in the DAFF and the vessel diameter.

**Figure 6:**
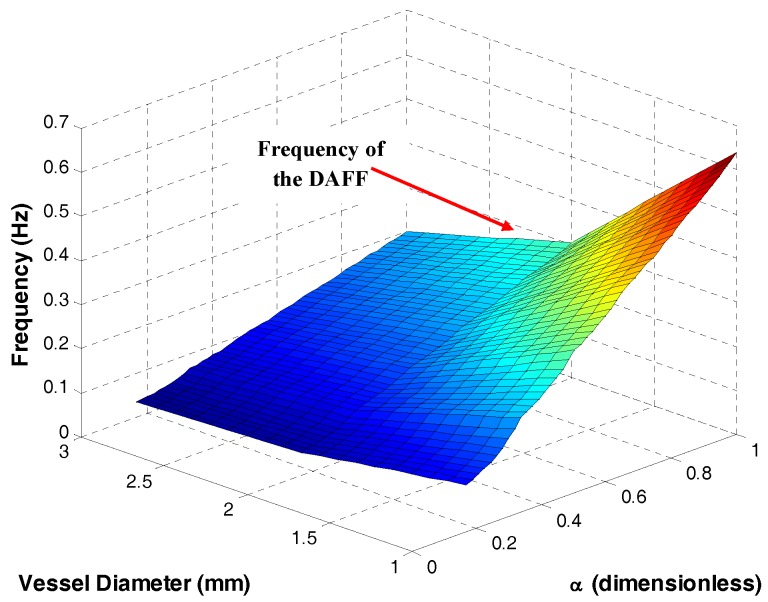
Frequency and asymmetry ratio α for high targeting performance in bifurcations of different diameters.

Figure 6 shows the relation between the frequency and asymmetry ratio (α), on the basis of the flowchart in Figure 5. Using the frequency and asymmetry ratio in Figure 6 for the DAFF, 100% guidance was achieved in the simulation. In addition, Figure 6 indicates that, for lower asymmetry ratios (α), a lower frequency can be applied, and the frequency increases with the rise in the asymmetry ratio. A high frequency does not provide sufficient guidance or delivery performance [27,34]. Therefore, a low asymmetry ratio (α=0.133, 0.33) and low frequency were used in this study.

### 2.3. In Vitro Study of Guidance of MNPs in a Y-Shaped Channel

**Figure 7:**
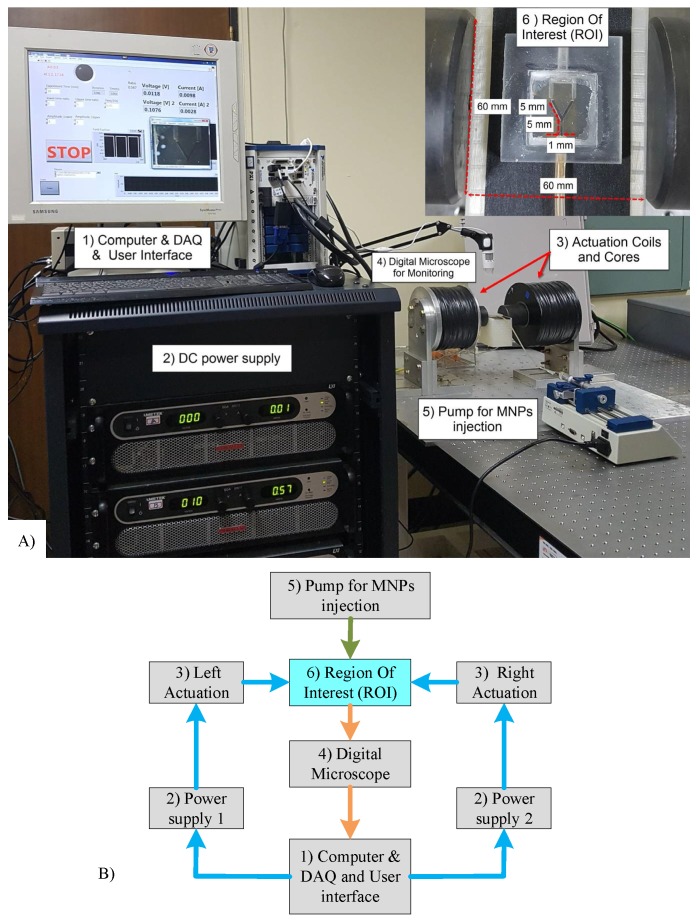
(**A**) The electromagnetic actuator comprises two coils (5000 turns and diameter of wire of $d_{w}$ = 1.0 mm) with two cores to increase the magnetic field density (cobalt–iron alloy VACOFLUX 50, VACUUMSCHMELZE, German); the cores are 19.5 cm in length and 6 cm in diameter. Two power supplies (AMETEK SGA 600/17, 10 kW) were utilized to generate currents of up to 6 A (gradient field strength of 2.8 T/m) in the experiments. (**B**) Schematic of the system.

The experimental setup of the magnetic actuation platform is shown in Figure 7. Electromagnetic actuators were designed to generate an adequate magnetic force to steer MNPs within the region of interest.

**Figure 8:**
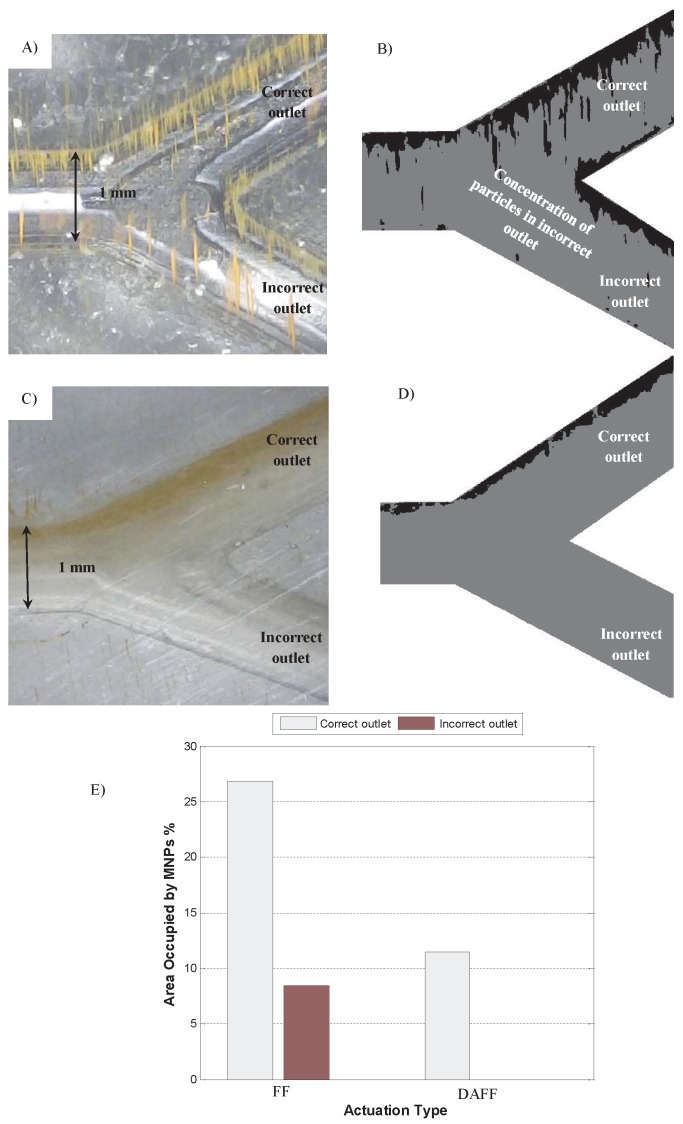
Video image analysis of the steering performance of a Y-shaped bifurcation. (**A**) Raw image for a field function (FF) of 6 A and 0.5 Hz. (**B**) Combined images obtained from averaging several hundred frames (6 A and 0.5 Hz). (**C**) Raw image under discontinuous asymmetrical field function (DAFF) of 6 A, 0.14 Hz and α=0.16. (**D**) Combined images obtained from averaging several hundred frames (6 A, 0.14 Hz and α=0.16). (**E**) The percentage of accumulated nanoparticles (NPs) in the correct and incorrect outlets under FF and DAFF.

Figure 8 shows the video image data for steering performance in FF and DAFF experiments, with the aim of providing a qualitative understanding of the different guidance behaviors of the FF and DAFF.

Aggregated particles move through the channel, as shown in the supplementary video. The aggregates are oriented along the direction of the main magnetic field and move with the flow. In the absence of a magnetic field, particles flow similarly through both outlets. As the magnetic field with FF (0.5 Hz, 6 A) is applied, the aggregates move toward the correct outlet. A sudden change in the magnetic field results in aggregates entering the incorrect outlet (Figure 8A; supplementary video). Some aggregates accumulate at the branch of the channel and at the boundary of the vessel; these form stationary aggregates.

Figure 8B shows combined images obtained by averaging multiple video frames. These plots show aggregate accumulation during the steering experiments. The black parts of the images represent areas with the highest density of aggregates. Consistent with the simulation platform described in the previous section, these results represent the scenario in which the guidance performance of the aggregates fluctuates under the FF and many particles enter the incorrect outlet. The formation of rod-shaped aggregates is also verified in this image. This phenomenon can be observed in Figure 8B. Although a pulsed magnetic field reduced blood clotting compared with a constant magnetic field [20,30], stationary aggregates and aggregates entering the wrong outlet can lead to blood clotting. Therefore, the DAFF is proposed to prevent blood clotting caused by aggregates.

As the magnetic field with the DAFF (0.144 Hz, α = 0.16) is applied, the aggregates move smoothly toward the correct outlet (Figure 8C and supplementary video). The size of the aggregates is reduced significantly, and stationary aggregates do not appear. Figure 8D shows combined images. Black parts of the images represent areas with the highest density of MNPs. These results confirm that the DAFF improves guidance of the MNPs. The DAFF also reduced the size of the aggregates.

Three differences between Figure 8B,D are evident. The size of the aggregates was reduced by the use of the DAFF for steering, aggregate fluctuations and entry to the incorrect outlet were minimized, and there were no stationary aggregates in the incorrect outlet or at the boundary. Statistical analysis of the images in Figure 8B,D verifies these phenomena. Figure 8E shows that the accumulation of particles in the correct outlet was twofold greater than that with the DAFF, which indicates that the aggregates were larger. Moreover, particles were absent from the incorrect outlet with the DAFF, but for the FF, 8.5% of the incorrect outlet was covered with MNPs. These results can also be seen in Figure 8B,D and the supplementary movie. By eliminating stationary aggregates and reducing the aggregate size, the DAFF reduces the risk of blood clotting.

### 2.4. Realistic Model Simulation

To study the effects of the DAFF, a realistic 3D model was simulated in COMSOL. A special procedure was used to extract data from a magnetic resonance image (MRI) and create a computer-aided design (CAD) model [35]. The model was imported into COMSOL Multiphysics to assess the particle trajectory. The realistic model consists of one inlet and six outlets with different diameters.

The average inlet velocity was selected on the basis of a realistic blood velocity (10 mm/s), and the computational fluid dynamics (CFD) module of COMSOL was used to obtain the velocity profiles in all channels. Other parameters were included in the simulation using the values in Table 1. The experimental studies in [36] suggested that 30% of MNPs are single particles and that the magnetic actuation cannot guide them. In the simulation, 1000 particles of an 800 nm diameter were realized uniformly in the inlet, and their trajectories were recorded. To illustrate the aggregation effects, 30% of the particles were considered to be single particles and 70% were considered to be aggregates. On the basis of the aggregate geometry (Figure 2), the equal diameter was considered to be

(18) $$d_{eq}=\frac{\pi }{2}n_{1}n_{2}^{2}d^{3}$$

where $n_{1}$ and $n_{2}$ are simulation parameters for the geometry of rod-shaped aggregates (Figure 2), and *d* is the particle diameter. The equal radius for the aggregates was considered to match the experimental results in [31]. Guidance for delivering MNPs under the designed actuation was studied statistically, and the number of particles reaching each outlet was calculated using the trajectory module of COMSOL.

**Figure 9:**
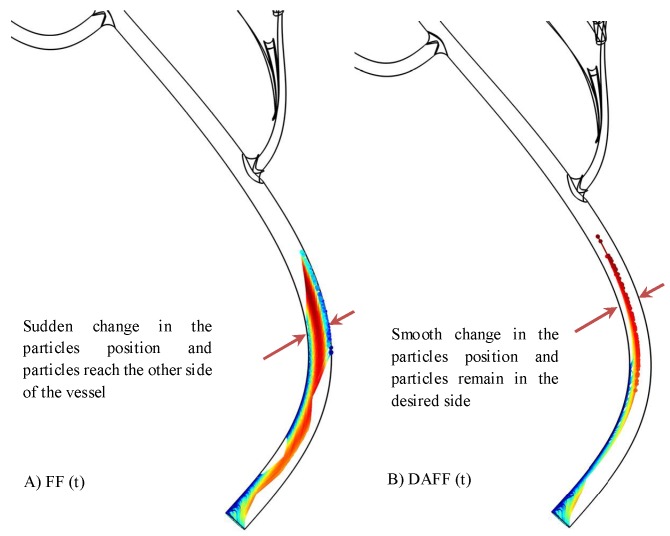
Particle tracking simulation of $T_{s}=7$ s for (**A**) a field function (FF(t)), and (**B**) a discontinuous asymmetrical field function (DAFF(t)).

To assess differences in the particle trajectories, the simulation time was initially considered to be $T_{s}=7$ s for both FF(t) and DAFF(t); the trajectories simulated are shown in Figure 9. Although the FF resolves the sticking issue during the movement of the MNPs, a sudden position change was observed, and the particles reached the opposite side, which led to MNP fluctuations within the vessel and limited the guidance performance. By contrast, the DAFF yielded smoother movements, which indicated a stable steering performance.

**Figure 10:**
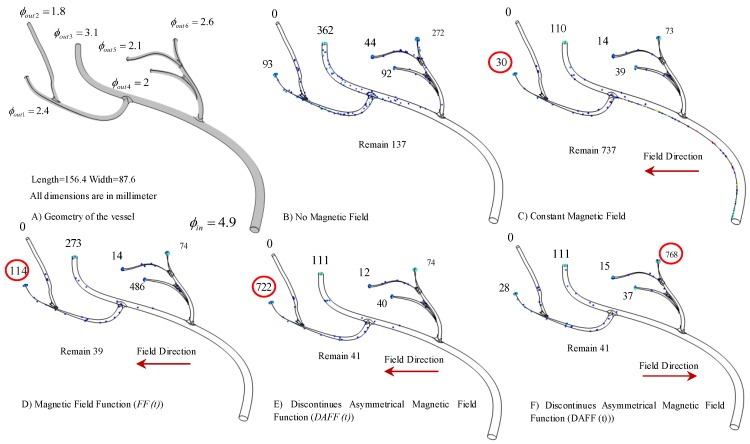
The simulation of the magnetic nanoparticles’ (MNPs’) distribution in a realistic vessel under the different designed actuation functions. The number close to each outlet is the number of particles reaching that outlet: (**A**) the realistic vessel geometry; (**B**) the distribution of particles under a drag force in the absence of a magnetic field; (**C**) a constant magnetic field; (**D**) the field function (FF); (**E**) the discontinuous asymmetrical FF.

Figure 10A shows the simulated vessel geometry. The effects of magnetic functions were studied in this simulation. Figure 10B shows that in the absence of a magnetic field, only 13.7% of the particles reached the targeted outlet, and particles were distributed on the basis of the drag force effects (Figure 10B shows the delivery performance). Figure 10C shows the effects of a constant magnetic field, which exacerbates the sticking issue; 73.7% of the particles remained within the vessel, and only 3% reached the outlet. By contrast, although the FF (6 A and 0.5 Hz) resolved the sticking issue and 3.9% of the particles remained in the vessel (Figure 10D), the number of particles in the targeted outlet increased only slightly to 11.4%. In comparison, for the DAFF with α = 0.166 and a frequency of 0.144 Hz (Figure 10E), the number of particles reaching the targeted outlet increased significantly (72.2% for the DAFF) as a result of the smooth movement of the particles in the vessel. By alternating the field direction, outlet 6 was also considered for delivery; using the same DAFF in this direction, 76.8% of the particles were delivered.

### 2.5. Passage of the BBB

In [21], the passage of NPs through blood vessels under a magnetic force was confirmed in vivo. The BBB being crossed under a magnetic field inside vessels using atomic force microscopy (AFM) was shown. In this section, we show the particles after crossing the BBB under a magnetic field, which is in agreement with the results in [21,27].

Superparamagnetic iron oxide (SPIO) NPs satisfy the conventional cytotoxicity assessment, but once they are exposed to a static magnetic field, their aggregation adversely affects their toxicity [37]. However, the coating reduces both the aggregation and toxicity of the NPs [38]. One of the main objectives of our paper was to reduce aggregation by using discontinuous asymmetrical magnetic actuation. Therefore, the combined effects of coating and the DAFF have a positive effect on reducing toxicity.

Pulse-shaped magnetic fields enhance passage through the BBB [26]. However, the functionality is limited by unmodeled aggregation. The in vitro experiments in this paper have showed the destructive effect of aggregation. Therefore, the DAFF is designed to disaggregate the MNPs and improve the delivery of NPs to the brain. To examine the effects of the proposed actuation scheme in vivo, several experiments were conducted using the experimental setup in Figure 7, and test subjects were positioned inside the region of interest (ROI) (Figure 1).

**Figure 11:**
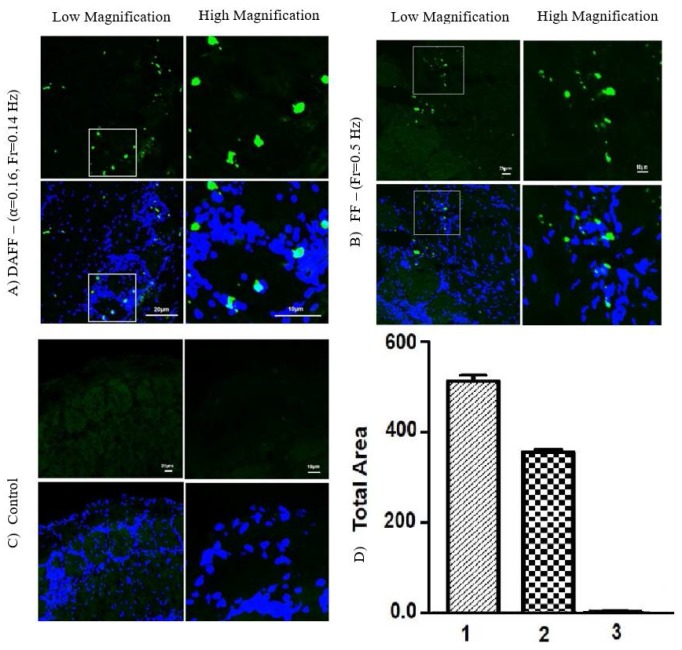
Confocal microscopy images of brain tissue samples: (**A**) DAFF(t) of 6 A, α = 0.16 and 0.144 Hz; (**B**) FF(t) of 6 A and 0.5 Hz [27]; (**C**) control; and (**D**) magnetic nanoparticle (MNP) accumulation. Data are the standard error of the mean (±SEM) of triplicate experiments (n=3): (1) DAFF(t) of 6 A, α = 0.16, and 0.144 Hz; (2) FF(t) of 6 A and 0.5 Hz; and (3) control.

Fluorescent MNPs (FMNPs) were injected into mice via the tail vein and then exposed to magnetic field conditions for 10 min. FMNP uptake in the brain was verified using confocal microscopy. Figure 11 shows confocal microscopy images of mouse brains under the DAFF (Figure 11A), the FF (Figure 11B), and no magnetic field (Figure 11C). We first analyzed the brains of mice in the absence of a magnetic field; no accumulation of FMNPs in the brain was observed (Figure 11C). FMNP uptake and transport were significantly higher for all magnetic actuations (Figure 11D, 1–2) compared with the control group (Figure 11D, 3). Interestingly, compared with the best condition for the FF, which was introduced in [27], the rate of particle transport across the BBB was increased significantly (1.5-fold) under condition 1 (DAFF, α = 0.16), which showed that a DAFF improved FMNP uptake and transport to the mouse brain compared with a FF (Figure 11D).

**Figure 12:**
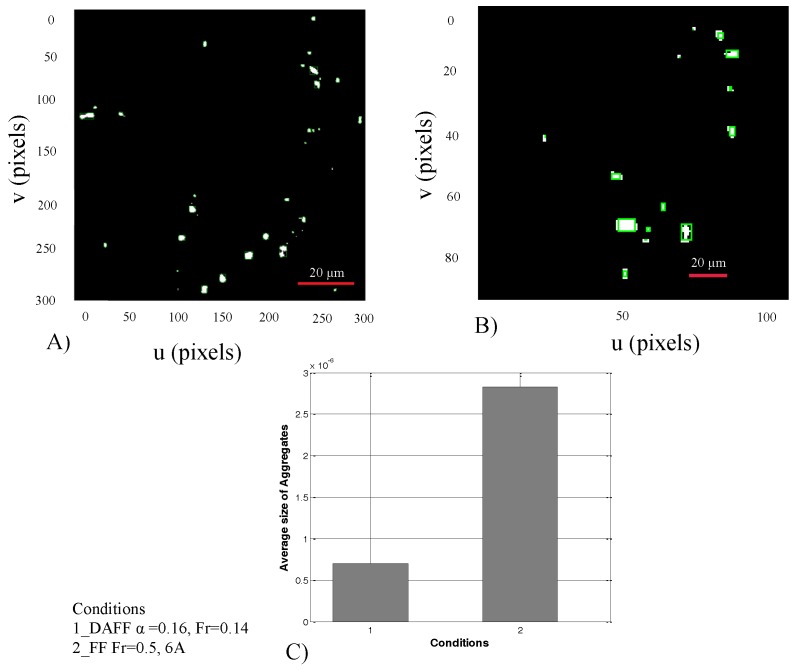
Aggregation of fluorescent magnetic nanoparticles (FMNPs) after crossing the blood–brain barrier (BBB) with different magnetic field functions (FFs). Aggregates under (**A**) discontinuous asymmetrical field function (DAFF), and (**B**) field function (FF); (**C**) MNP accumulation under (1) DAFF(t) of 6 A, α = 0.16 and 0.144 Hz; and (2) FF(t) of 6 A and 0.5 Hz.

Figure 12 shows the average size of MNPs in the brain after crossing the BBB. Figure 12A,B illustrates particle detection and categorization after BBB crossing under the DAFF and FF. Figure 12B shows the average size of aggregates. Aggregates in condition 2 (FF) had a larger average size than those in condition 1 (DAFF). Therefore, a DAFF can be used to enhance the transport of MNPs across the BBB, while resulting in markedly smaller aggregates. More importantly, our results demonstrate the importance of exploring the effects of variation in the magnetic field in the context of in vivo drug delivery applications.

## 3. Experimental Section

### 3.1. System Setup

The region of interest is 60 mm in diameter at the center of the actuation system. The electromagnetic actuator comprises two coils (5000 turns; wire diameter of $d_{w}$ = 1.0 mm) with two cores to increase the magnetic field intensity (cobalt–iron alloy VACOFLUX 50; VACUUMSCHMELZE, Hanau, Germany); the cores are 19.5 cm in length and 6 cm in diameter. Two power supplies (SGA 600/17, 10 kW; AMETEK, Berwyn, PA, USA) are used to generate currents of up to 17 A (maximum gradient field strength of 7.9 T/m). In the experiments, a maximum current of 6 A (2.8 T/m) was used [34]. An NI PXIe 8135 is used to control the coils, and a digital microscope is used to monitor the MNPs.

### 3.2. In Vitro Study

To assess the DAFF experimentally, magnetic silica particles (SiMAG-Silanol, 750 nm diameter; Chemicell GmbH, Berlin, Germany) were guided within a Y-shaped channel using the proposed dynamic magnetic actuation. A Y-shaped channel with a length of 5 mm and diameter of 1 mm, with an equal stream flowing in both outlets, was used to study the guidance performance.

### 3.3. In Vivo Study

Fluorescent carboxyl magnetic Nile Red particles (FMNPs) (1% *w/v*; CATALOG NO: FCM-02556-2) were purchased from Spherotech (Libertyville, IL, USA). The NPs were 0.20–0.39 μm in diameter and were used in a previous study of drug delivery to the brain [27]. Nile Red is polymerized inside the core of the beads during the manufacturing process. In brief, the bead is polymerized in the first step with the Nile Red, magnetite, and styrene. The fluorescent tag is attached with NPs (FMNPs); the excitation spectra of these FMNPs ranged from 400 to 500 nm, and they showed highly efficient fluorescence in the Fluorescein isothiocyanate (FITC) channel at 488 nm when observed under a confocal laser scanning microscope (FLUOVIEW FV1000; Olympus, Tokyo, Japan) with an argon ion laser. The power of the laser was 20%. During the confocal microscopy experiment, we used DAPI (6-diamidino-2-phenylindole) dye to label the nuclei of the brain cells. To trace the FMNPs inside the mouse brain, the dye was attached to the magnetic particles before injecting them into the mouse; thus there was no need for additional staining with the same dye to trace these particles inside the brain. The FMNPs were traced inside the brain with the help of the fluorescent molecules already attached to the magnetic particles; these were visible in the FITC channel at 488 nm (Figure 11).

Male wild-type C57BL/6N mice (25–30 g, 8 weeks old) were purchased from Samtako Bio (Gyeonggi-do, South Korea). The mice were acclimatized for 1 week in the university animal house under a 12 h/12 h light/dark cycle at 23 ${}^{\circ }$C and 60% humidity, and they were provided with food and water ad libitum. The mice were divided randomly into the following groups: (A) *DAFF*(*t*) of 6 A, α = 0.16 and 0.144 Hz; (B) *FF*(*t*) of 6 A and 0.5 Hz; (C) control. The mice in groups A and B received 0.4 mL of FMNPs via intravenous (i.v.) injection and were then exposed to the magnetic field for 10 min. The control animals were given 0.4 mL of i.v. 0.9% saline solution. The mice were euthanized following the treatments. All efforts were made to minimize the number of mice used and their suffering. The experimental procedures were approved by the Animal Ethics Committee of the Division of Applied Life Sciences, Department of Biology, Gyeongsang National University, South Korea. Brain tissues from all of the groups were collected after the treatments. Transcardial perfusion was performed with 1× phosphate-buffered saline (PBS) followed by 4% ice-cold paraformaldehyde. The brain tissues were post-fixed overnight in 4% paraformaldehyde and then transferred to 20% sucrose until they sank to the bottom of the tube. The brains were frozen in an optimum cutting temperature (OCT) (Tissue-Tek O.C.T. compound; Sakura Finetek USA, Torrance, CA, USA) and then cut into 14 μm sections in the coronal plane with a CM 3050S cryostat (Leica, Wetzlar, Germany). The sections were thaw-mounted on Probe-On positively charged slides (Thermo Fisher Scientific, Waltham, MA, USA) and stored at −70 ${}^{\circ }$C.

The brain tissue slides were dried overnight and then washed twice with 0.01 M PBS for 15 min each. The tissue sections were stained with DAPI for 10 min and rinsed with PBS, and glass coverslips were mounted on the slides with a fluorescent mounting medium. Images were captured using a confocal microscope (FLUOVIEW FV 1000; Olympus).

## 4. Conclusions

A novel magnetic FF design that can minimize aggregation effects is proposed. The proposed DAFF was simulated by a computational platform to study the targeting performance in a Y-shaped vessel. A DAFF was designed to achieve a guidance performance of 100%. Then, we showed experimentally that the proposed DAFF can increase the delivery performance via steering at the bifurcation in vitro. The size of aggregates is also reduced in comparison with the FF. Furthermore, stationary aggregates are absent in the presence of a DAFF. In vivo experiments also revealed the effectiveness of the DAFF in terms of BBB passage. Image analysis reveals that, compared with the FF, the DAFF results in the generation of smaller aggregates after the passage of the BBB. The DAFF (6 A, α = 0.16, and 0.144 Hz) performed the best for BBB passage and drug uptake. The new actuation scheme, which was examined experimentally for MNP guidance and passage of the BBB, shows promising results. The mechanism of BBB passage and the determination of the optimum actuation function to enhance BBB passage should be the subjects of future work.

## Figures and Tables

**Table 1:** Simulation parameters.

Parameter	Value
Particle density	6450 kg/m${}^{3}$
Particle diameter	800 nm
Blood density	1050 kg/m${}^{3}$
Blood viscosity	0.004 Pa·s
Air relative permeability	1 (dimensionless)
Blood relative permeability	1 (dimensionless)
Blood temperature	293.15 K

**Table 2:** Units for magnetic properties.

Symbol	Quantity	Value
$L_{v}$	Vessel length	10 mm
$T_{c}$	Normal exit time	5 s
$R_{ve}$	Vessel elongation	20, 10, 6.6, 0.5 (dimensionless)
$R_{f}$	Force Factor	0.3,0.6,0.9,1.2,1.5,1.8 pA·m

## Supplementary Materials

The supplementary video is available online at http://www.mdpi.com/2079-4991/8/1/3/s1.
